# Assessing the impact of paternal emigration on children ‘left-behind’—A cohort analysis

**DOI:** 10.1016/j.jmh.2025.100308

**Published:** 2025-01-25

**Authors:** Natalia Norori, Lucy Barrass, Maria Theresa Redaniel, Nanette R. Lee, Laura D. Howe, Duleeka Knipe

**Affiliations:** a Population Health Sciences, Bristol Medical School, University of Bristol, Bristol, UK; b NIHR ARC West, Population Health Sciences, University of Bristol, Bristol, UK; c USC-Office of Population Studies Foundation, Inc., University of San Carlos, Cebu City, Philippines; d South Asian Clinical Toxicology Research Collaboration, Faculty of Medicine, University of Peradeniya, Peradeniya, Sri Lanka

**Keywords:** Migration, Mental health, Education, Determinants of health

## Abstract

**Background:**

Previous work has shown that children ‘left-behind’ as a consequence of parental migration experience worse outcomes, although the majority of this evidence focuses on short- rather than long-term effects.

**Methods:**

Using data from the Cebu Longitudinal Health and Nutrition Survey cohort (n = 1651), we assessed the association of paternal emigration (identified based on evidence of remittances sent back by mother's spouse) during childhood with the mental health and educational attainment at age 18 of Filipino children, adjusted for sex, socioeconomic position and paternal education. We explored whether timing of emigration, and household composition modified associations observed.

**Findings:**

Children who had migrant fathers were found to be 1.24 times more likely to have high educational attainment at age 18 than children who did not have migrant fathers, although the association was imprecise (95 % confidence intervals: 0.83-1.85). We found no statistical evidence of a difference between children who experienced paternal migration compared to those who did not in terms of depressive symptoms or suicidal ideation at age 18. There was evidence that experiencing paternal migration in older childhood (≥10 years) was associated with better mental health. We found evidence that household composition modified associations for depressive symptoms.

**Interpretation:**

This study does not suggest a detrimental long-term impact of paternal emigration on children ‘left-behind, either for educational attainment or mental health. This may reflect beneficial effects of paternal migration and/or pre-existing socioeconomic and health differences amongst families who do and do not experience paternal migration.

## Introduction

In recent years, labour migration has grown in complexity and scale. In 2022, there were around 281 million international migrants worldwide, and 169 million of them were migrant workers (International Organisation for Migration, 2022). Often migrant workers do not take their families with them, resulting in children remaining in their current place of residence while at least one parent is either overseas or elsewhere in the country of origin. Consequently, children with migrant parents may be exposed to sudden and prolonged changes in family dynamics during their childhood that change parent-child caring (Thenmozhi and Mohan, 2024; Castell Roldán and Alvarez Anaya, 2022).

Experiencing parental emigration has been previously associated with a wide range of outcomes. A study that analysed data from the Young Lives Project, an international study on childhood poverty following cohorts in four countries, suggests that experiencing parental emigration results in decreased cognitive and academic ability at the time of the migration experience (Viet Nguyen, 2016). A systematic review of 111 observational studies found that children with migrant parents had a higher risk of anxiety, depression, suicidal ideation, substance abuse and conduct disorder when compared to children with non-migrant parents (Fellmeth et al., 2018). Similarly, children with migrant parents have been shown to have poorer educational attainment than those living with both parents (Lu, 2014). Outcomes may differ depending on which parent is absent. Previous evidence suggests that children with migrant fathers have better educational and mental health outcomes than children with migrant mothers (Marchetta and Sim, 2021; Liu et al., 2009). Many of these studies focus on short term outcomes, with very little evidence of the long term impact on children ‘left-behind’. Initial distress from parental emigration is likely, but there is some evidence to suggest that this does not remain as the child gets older (Lei et al., 2021; Fu et al., 2023).

The impact of parental emigration can vary according to several contextual factors, including migration patterns, socioeconomic position (SEP), and household characteristics (Vanore et al., 2021). It can also differ depending on the age of the child at the time parental migration first occurred. For example, one study found that children who experience parental emigration during their early childhood have higher scores of anxiety and depression when compared to older children (Liu et al., 2009). Similarly, a study in China exploring height of ‘left-behind’ children found that those left at a younger ages had shorter stature than children whose parents emigrated at older ages (Zhang et al., 2015). These results indicate that children left at an earlier age may be more susceptible to negative outcomes of migration than their older counterparts. Being ‘left-behind’ in a nuclear household may also have different effects to experiencing parental emigration in a bigger household due to more adults being available in extended households to provide emotional and social support to the child, potentially mitigating the effects of an absent father or mother (Javier et al., 2018). Although migration is widely recognized as a social determinant of health, more data are needed to understand its health and psychosocial effects on children living in different contexts.

Labour migration has been a key feature of Southeast Asian economies since the 1980s, when the rise of international labour migration was first recorded. The Philippines continues to be a large producer of migrants in Asia, and receives the fourth largest amount of remittances globally (International Organisation for Migration, 2022). Remittances are sums of money sent back to families from the migrant workers abroad. The Philippines is one of the few countries with an institutionalized labour migration policy that encourages temporary labour migration and protects migrant workers and their families. The country has also enacted several laws that regulate the recruitment of Overseas Filipino Workers in order to protect them from forced labour and human trafficking. Furthermore, the Overseas Workers Welfare Association (OWWA) provides several services to migrant workers and their dependents, including family assistance loans, health and life insurance, and counselling (Department of Migrant Workers, 2024). The main driver of migration, both internal and international, for Filipinos is economic benefits through better employment opportunities with likelihood of increased wages that can be sent back to families, as well as better job security and positive working environments (Jolipa, 1980; Toyin-Thomas et al., 2023). Economic recessions and political instability in the 1980s may have been a determining factor for migration in the past, the COVID-19 pandemic induced recession is likely to have made more individuals migrate in recent years, and workers may migrate in coming years due to the impact of climate change on traditional jobs such as farming (UNANIMA International, 2022).

The institutionalization and acceptance of labour migration in the Philippines make it a unique environment to study the impact of experiencing parental emigration. There is no exact data on the number of children affected by labour migration, but it is estimated that 9 million Filipino children have at least one migrant parent (Dominguez and Hall, 2022). There is mixed evidence available for educational and mental health outcomes, and the evidence comes with some limitations (Dominguez and Hall, 2022). Firstly, the body of evidence consists of exploratory analysis and retrospective studies where migration was measured at only one point in time (Asis, 2006; Graham and Jordan, 2011; Lam and Yeoh, 2019). Secondly, the study samples were not representative of the Filipino population at large with some studying specific school or college groups which could be correlated with SEP or studying only migrant populations (Smeekens et al., 2012; Bernardo et al., 2018). Further research is required to understand how contextual factors influence the effects of parental emigration in the Philippines. Finally, much of the evidence is based upon qualitative studies which are unable to quantify the effects of paternal emigration.

We aimed to assess the long-term effects of paternal emigration on the mental health and educational attainment of Filipino children who were left behind.

By using data from the Cebu Longitudinal Health and Nutrition Survey (CLHNS), we aimed to answer the following questions: i) Is there an association between experiencing paternal emigration during childhood and depressive symptoms, suicidal ideation, and poor educational attainment during adulthood?; ii) Does household composition modify any of the associations?; and iii) Do any of the associations differ depending on the age of the child at time of earliest migration?

## Methods

### Study setting

The Philippines is a country situated in Southeast Asia. The World Bank classifies the Philippines as a lower-middle income country with a GDP per capita of US$3,498.5 (The World Bank, 2024); the Philippines ranks 7^th^ out of 11 on this measure in Southeast Asian countries. In 2023, it was estimated that 10.9 % of families were below the poverty threshold (Philippines Statistics Authority, 2024). Metropolitan Cebu is one of the largest metropolitan areas in the Philippines, with high density neighbourhoods, less dense peri-urban areas, rural towns and more isolated communities in the mountains and islands (Adair et al., 2011).

### Participants

CLHNS is an ongoing longitudinal birth cohort studying children born in Cebu between May 1983 and April 1984. A single stage random clustering sampling procedure was used to select urban and rural barangays, small administrative units forming the most local level of government in the Philippines, from metropolitan Cebu. All pregnant women in these areas were approached, via house-to-house interviews, for recruitment into the study, resulting in 3,327 eligible pregnant women completing the baseline survey. CLHNS has been previously described in more detail elsewhere; there were 3,080 live single births whom have been followed up at multiple different time points since (Adair et al., 2011). At initiation, the cohort was representative of the demographic and socioeconomic diversity of the area. Participants in this analysis were excluded if they did not have data on the outcomes at age 18, or did not have date of birth, sex or household type at baseline. Participants were also excluded if they did not live with their mother to ensure we were capturing the emigration of the father of the child. Ethical approval was not obtained for the secondary analysis of this data, however, all surveys and data protections were carried out in accordance with The Code of Ethics of the Declaration of Helsinki, with IRB approvals obtained from the University of North Carolina at Chapel Hill, and the University of San Carlos Research Ethics Committee, Cebu, Philippines.

### Measures

#### Paternal emigration

During the baseline and follow-up surveys, CLHNS mothers were asked the following yes/no question: “ Do you receive remittances from spouse abroad as another source of income for the household?”. Our study assumes that when the mother answered yes to this question, the cash remittances received were sent by the children's father. Data on remittances was recorded in 1983, 1985, 1986, 1991, 1994 and 1998. Paternal emigration was coded as yes if remittances were reported at one or more time points.

Survey date and date of birth of the child were used to calculate age at the time paternal emigration was first recorded. Using this information participants were categorized into the following age groups: Never experienced paternal emigration, experienced paternal emigration at ages 0-9, experienced paternal emigration at ages 10-15.

#### Educational attainment

Participants were asked to provide information on the highest level of education they had completed at age 18. These data were collected in 2002, when the Philippines educational system consisted of 6 years of elementary school and 4 years of high school; in this cohort, education was free up to the completion of high school but some may have entered into the paid, private education system. By age 18, the Filipino children should have completed high school if they had followed the standard schedule, although only primary school was mandatory. Considering this, educational attainment was recorded to a dichotomous variable with two categories: “Completed high school; has not completed high school”.

#### Depressive symptoms

Depressive symptoms were assessed at age 18, using a scale created based on a modified 16-item version of the Center for Epidemiologic Studies-Depression Scale (CES-D). The children were asked how often in the past 4 weeks they had experienced a series of symptoms, such as: feeling lonely, feeling worried, feeling worthlessness, feeling disliked, having difficulty sleeping, feeling hopeful, and feeling happy. Each question was given a score of 0, 1 or 2 (“none of the time”=0, “occasionally”=1, “most of the time”=2), with reverse coding for positive items e.g. felt happy. Scores were summed and a cut off of one standard deviation above the mean was used to determine if individuals were experiencing higher levels of depressive symptoms; in this instance, the cut-off was scores greater than or equal to 12. The mean and SD value were used as there is no validated cut off for this modified scale and previous analyses using different data have used this method (Puyat et al., 2021; Assessment of depression in adolescents using the Center for Epidemiologic Studies Depression Scale [press release], 1990). The cutoff of 12 is also aligned with the proportion used for CES-D-10 cutoff, when taking into account the three-item response. The internal consistency reliability of the sample was found to be acceptable for our analysis (Cronbach's alpha = 0.69).

#### Suicidal ideation

Suicidal ideation was measured at age 18, as part of the CES-D. Young adults with suicidal ideation do not necessarily show symptoms of depression (Puyat et al., 2021). For this reason, we decided to assess suicidal ideation and depressive symptoms separately. Participants were asked the following questions: “In the past 4 weeks, did you wish you were dead?”; ‘‘In the past 4 weeks, did you have the idea of taking your own life?”. Suicidal ideation was coded as yes if participants answered positively to any of the two questions.

#### Modifying factors

Household composition, whether the child lived in a nuclear household or otherwise, was investigated as a modifier of the relationship between paternal emigration and the three outcomes. To measure household composition, information on household type at time of emigration was used for participants who had experienced parental emigration, and at baseline for those who had not, to create a dichotomous variable that recorded if participants lived in a single nuclear household (e.g. mother with spouse, with/without children), or in any other type of extended household (e.g. grandparents, aunts, uncles -mother included in household).

#### Covariates

Data on participant's sex, assets ownership and paternal education were obtained from the questionnaire completed at baseline. To measure household SEP, a wealth index was created including the following variables: air conditioner ownership, car ownership, house ownership, television ownership, tape recorder ownership, refrigerator ownership, ownership of other appliances, household size, type of household and house construction material. To create the index, Principal Components Analysis (PCA) was used, with the first component being extracted and turned into quintiles to use as a measure of SEP. Asset ownership is unlikely to change in response to short-term income variations, therefore, an assets-based wealth index is considered a good long-term estimate of SEP (Howe et al., 2008). The weights for each variable included in the PCA are shown in supplementary Table S1. Paternal education spanned from having no grades completed to having a postgraduate degree.

### Analysis

Descriptive statistics were calculated, and a series of multivariable logistic regression models were fitted to assess the relationship between paternal emigration and educational attainment, depressive symptoms and suicidal ideation; each model was estimated independently. First, the relationship between paternal emigration and the three outcomes was explored, without adjusting for any covariates and then, a second model adjusted for sex, SEP and paternal education at baseline. To further explore the association and to determine if age at time of migration had any effect, the initial analysis was repeated using this as the exposure. Associations between the exposure and outcomes are presented stratified by household composition at baseline. To assess if household composition was modifying the associations seen, we fitted the model with and without the interaction, and used a likelihood ratio test to compare the models. Post-hoc, we additionally stratified the primary analysis by sex to determine the impact of this, adjusting for SEP. We conducted a complete case analysis and explored whether there were any differences between the baseline characteristics of those included within this analysis compared to those excluded.

## Results

### Descriptive statistics

Of the 3327 mothers in the CLHNS, 3080 had a single live birth and 1,651 children had complete data for the variables of interest and were included in this analysis (Figure S1). The baseline characteristics of participants included in the study were different to those excluded from the study apart from when looking at sex (Table S2). Those excluded had higher mean scores for paternal education and wealth index, were more likely to experience paternal emigration at baseline, and less likely to be living in one nuclear family.

Study characteristics of the cohort are shown in Table 1. 9.6 % of the children in the study sample experienced paternal emigration between 1983 and 1998. Amongst study participants, 52.3 % were male, and 65.4 % lived in a single nuclear family household. 61.4 % had completed high school by age 18, 14.0 % had higher levels of depressive symptoms, and 15.8 % reported having suicidal ideation.

**Table 1:** Characteristics of the study sample for key variables.

	Children of migrant fathers N (%) n = 159	Children of non-migrant fathers N (%) n = 1492	Total N (%)
**Male**	93 (58.49)	770 (51.61)	863 (52.27)
**Household type**			
One nuclear family	89 (55.97)	991 (66.42)	1080 (65.41)
**Wealth index**			
Quintile 1 (lowest SEP)	27 (16.98)	356 (23.86)	383 (23.10)
Quintile 2	15 (9.43)	313 (20.98)	328 (19.87)
Quintile 3	25 (15.09)	286 (19.17)	310 (18.78)
Quintile 4	31 (19.50)	290 (19.44)	321 (19.44)
Quintile 5 (highest SEP)	62 (38.99)	247 (16.55)	309 (18.72)
**Age at time of first paternal emigration**			
Never experienced paternal emigration	-	1492 (100)	1492 (90.37)
Paternal emigration at ages 0-9	81 (50.94)	-	81 (4.91)
Paternal emigration at ages 10-15	78 (49.06)	-	78 (4.72)
**Educational attainment**			
Completed high school	117 (73.58)	897 (60.12)	1014 (61.42)
**High depressive symptoms**	12 (7.55)	219 (14.68)	231 (13.99)
**Suicidal ideation**	18 (11.32)	242 (16.22)	260 (15.75)

### Paternal emigration and educational attainment

**Fig. 1:**
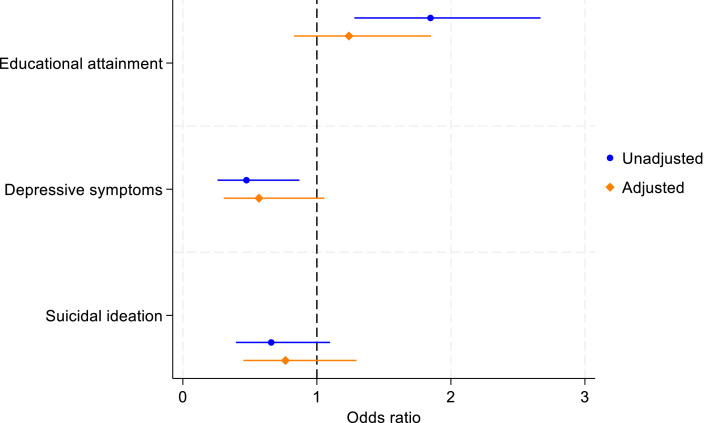
Unadjusted and adjusted associations of paternal emigration during childhood and outcomes at age 18.

In unadjusted analysis, children who experienced paternal emigration were nearly twice as likely to complete high school by age 18 (OR =1.85, 95 % confidence interval (CI) 1.28-2.67), when compared to those who did not experience paternal emigration (Fig. 1). The observed direction of association persisted after adjusting for sex, SEP and paternal education but the association was attenuated towards the null (OR 1.24, 95 % CI 0.83-1.85).

The association was further explored by looking at age at time of migration. Point estimates suggested children who experienced paternal emigration during their early years of childhood had increased odds of completing high school by age 18, when compared to those who never experienced paternal emigration (Table 2). The odds were found to be higher among children whose fathers migrated when they were between 10-15 years of age. When adjusted for sex and SEP, the direction of associations remained the same for the older children, but reversed for the younger group with the latter not supported by statistical evidence.

**Table 2:** Unadjusted and adjusted associations of age at time of paternal emigration during childhood and outcomes at age 18.

Age at time of paternal emigration	Unadjusted	Adjusted for sex and SEP
	*OR (95 % CI)*	*OR (95 % CI)*
**Educational attainment**		
No paternal emigration	1.00	1.00
Paternal emigration at ages 0-9	1.40 (0.87-2.26)	0.71 (0.42-1.20)
Paternal emigration at ages 10-15	2.57 (1.47-4.50)	2.21 (1.22-4.00)
**Depressive symptoms**		
No paternal emigration	1.00	1.00
Paternal emigration at ages 0-9	0.73 (0.36-1.47)	0.94 (0.45-1.96)
Paternal emigration at ages 10-15	0.23 (0.07-0.74)	0.26 (0.08-0.84)
**Suicidal ideation**		
No paternal emigration	1.00	1.00
Paternal emigration at ages 0-9	0.81 (0.42-1.56)	0.99 (0.50-1.95)
Paternal emigration at ages 10-15	0.51 (0.23-1.12)	0.57 (0.26-1.26)

### Paternal emigration and depressive symptoms

Our study found weak evidence of an association between paternal emigration and decreased symptoms of depression at age 18, with the association attenuating in adjusted analyses (Fig. 1).

The association was further explored by looking at age at migration (Table 2). Children aged 0-9 at time of first exposure were at a lower likelihood of developing depressive symptoms, although the CI crossed the null value, and the OR was close to one once confounders were adjusted for (OR 0.94, 95 % CI 0.45-1.96). Children first experiencing paternal emigration aged 10 and above had lower odds of developing depressive symptoms at age 18, compared to children with no paternal emigration. The association remained after adjusting for covariates (OR 0.26, 95 % CI 0.08-0.84).

### Paternal emigration and suicidal ideation

There was no evidence of an association between paternal emigration during childhood and suicidal ideation at age 18 (Fig. 1). When exploring by time of paternal emigration, children who first experienced paternal emigration at older ages had lower odds of ideation (Table 2), but associations were estimated with high degree of imprecision.

### Household composition as an effect modifier

There was some statistical evidence (via likelihood ratio tests) that the association between paternal emigration and any of the three outcomes was modified by the children's household composition (Table 3). In particular, the association between paternal migration and better mental health (both depression and suicidal ideation) was stronger in children living in extended families.

**Table 3:** Household composition stratified associations of paternal emigration during childhood and educational attainment, depressive symptoms, and suicidal ideation at age 18.

	Unadjusted			Adjusted		
Children with paternal migration vs without	One nuclear family	Any form of extended family		One nuclear family	Any form of extended family	
	*OR (95 % CI)*	*OR (95 % CI)*	*p-value for interaction*	*OR (95 % CI)*	*OR (95 % CI)*	*p-value for interaction*
**High educational attainment**	2.08 (1.28-3.41)	1.48 (0.85-2.58)	0.37	1.31 (0.76-2.25)	1.14 (0.61-2.12)	0.66
**Depressive symptoms**	0.68 (0.35-1.35)	0.20 (0.05-0.84)	0.09	0.90 (0.44-1.83)	0.21 (0.05-0.91)	0.07
**Suicidal ideation**	0.80 (0.42-1.50)	0.49 (0.21-1.18)	0.37	0.97 (0.50-1.88)	0.45 (0.18-1.15)	0.33

Stratified analyses for sex can be seen in Table 4. There were few differences in the association of paternal migration with depressive symptoms and suicidal ideation between sexes, however, paternal emigration is more strongly associated with better educational attainment in males compared with females overall.

**Table 4:** Sex stratified associations of paternal emigration during childhood and educational attainment, depressive symptoms, and suicidal ideation at age 18, adjusted for socioeconomic position.

	Educational attainment		Depressive symptoms		Suicidal ideation	
	*Male*	*Female*	*Male*	*Female*	*Male*	*Female*
**Paternal emigration**						
Experienced paternal emigration (v. no paternal emigration)	1.52 (0.93-2.51)	0.87 (0.45-1.67)	0.53 (0.21-1.38)	0.61 (0.27-1.40)	0.73 (0.32-1.6)	0.80 (0.40-1.60)
**Age at time of paternal emigration**						
No paternal emigration	1.00	1.00	1.00	1.00	1.00	1.00
Paternal emigration at ages 0-9	0.84 (0.44-1.61)	0.55 (0.23-1.34)	0.99 (0.33-2.92)	0.95 (0.35-2.58)	0.90 (0.31-2.67)	1.07 (0.44-2.58)
Paternal emigration at ages 10-15	2.91 (1.34-6.15)	1.34 (0.52-3.36)	0.19 (0.03-1.41)	0.33 (0.08-1.41)	0.59 (0.18-1.94)	0.56 (0.19-1.64)

## Discussion

To the best of our knowledge, this is the first cohort analysis to investigate the long-term effects of paternal emigration at childhood on the mental health and educational attainment of Filipino children at age 18. Our findings suggest that experiencing paternal emigration during childhood is associated with a higher level of educational attainment, especially among males. For both suicidal ideation and depressive symptoms, point estimates were suggestive of a lower likelihood in children with paternal emigration, but confidence intervals were wide and included the null. Whilst associations remained in both those who experienced first paternal migration at 0-9 and ≥10 age categories with education, there were some differences for depressive symptoms, and those in the ≥10 age category had CI that did not cross the null, suggesting a protective effect of paternal emigration for that group.

We found this population to have higher levels of depressive symptoms than a study in a similar age group on the Philippines nationally, several years after our study period, with no general population data available for 2002 (Puyat et al., 2021). Our results are consistent with previous evidence from the Philippines around migration and outcomes. A study that used 2003 nationwide data reported that children with migrant parents are more likely to have higher academic performance, when compared to children with non-migrant parents, similar to this current study (Asis, 2006). The beneficial effects of paternal migration on education can be attributable to the increase in family resources due to remittances sent back, resulting in increased ability to buy food and housing security; we found 37.0 % of our sample who had experienced paternal emigration had increased their SEP, measured through the same wealth index used throughout. Paternal emigration may also allow families to afford private or higher education for their children, encouraging a longer time spent in education whereas those without the additional income may not be able to do so. Similarly, a different study showed that the psychosocial well-being of children from the Philippines was not affected by parental emigration, which is broadly consistent with our findings not stratified by age at emigration (Graham and Jordan, 2011). This may be due to the increase in income allowing families to reside in safer, more stable environments which may influence better mental health and well-being. Another possible explanation is that selection into migration is greater in families with higher initial SEP. In this study, families who had experienced paternal migration at baseline or who went on to experience it had a higher mean wealth index score at baseline (p<0.001) than those who did not, and associations were attenuated with adjustment for socioeconomic indicators, indicating that this selection into migration could be driving some of the beneficial associations seen.

However, the results of our study differ from what has been previously reported in other settings. A study using data from the Young Lives cohort conducted in Ethiopia, India, Peru, and Vietnam, suggested that parental emigration had a harmful effect on the health and cognitive skills of children in the cohort (Viet Nguyen, 2016). The study examined the effect of paternal and maternal emigration separately, and found that in three of the four countries, paternal emigration was associated with lower children's educational test scores, although results from Vietnam aligned with the direction of our findings; most of these findings were not supported by statistical evidence (Viet Nguyen, 2016). Evidence from Africa suggests children who experience paternal emigration have a higher risk of suffering psychological distress than children without a migrant parent, although results aren't as severe as when the mother is absent through migration (Mazzucato et al., 2015). Our results may differ from the literature presented above as they focus on the short-term impact of paternal emigration, where you would expect some increases in psychological distress due to the disruption a father moving away is likely to cause. When looking at studies that explore the longer-term impacts, they also find no longer-term impacts of poorer mental health, consistent with our results (Lei et al., 2021; Fu et al., 2023). The increases in educational attainment long-term indicate that paternal emigration does have benefits to children ‘left-behind’ and although it may cause short-term distress, the benefits on their future may outweigh the initial downsides.

The differences between our findings and findings from settings outside of the Philippines could also be influenced by the Philippines’ migration governance framework. The country has more than four decades of experience designing and implementing policies that support and protect the rights of Overseas Filipino Workers. It also included migration in their development agenda (National Economic and Development Authority, 2021). The normalization of labour migration may reduce the traumatic effect of parental separation as children are more likely to understand what is happening as it isn't an unfamiliar occurrence in their peer circles and also there may be better communication strategies in place for contacting and maintaining relationships with the migrated parent (Graham and Jordan, 2011; Lu, 2018). Normalisation also reduces the traumatic effects due the increased support received from the government which ensures the children who remain are protected. Additionally, the Philippines has a long history of labour migration and is not only accepted, but often desired in some respects (Ang et al., 2023).

Similar to other studies that explored the impact of age at parental migration, we found that children fared better on all three outcomes when they first experienced paternal emigration after the age of 10 (Veling et al., 2011; Zhang et al., 2015). Younger children may not see this effect as younger children may be more dependent on their parents than adolescents and may require higher levels of social support from parental networks in those early years (Eisenberg and Morris, 2002).

Most research looking at the impact of parental emigration has been conducted in countries that offer low levels of support to migrant workers and their dependents, whereas the Philippines provides a wide range of support including post-arrival orientation seminars, tailored to specific countries, making it a unique study setting (Ang et al., 2023). A strength of this study is that it is the first cohort analysis to provide a broad picture of the impact of paternal emigration, and to quantify its long-term effects in a country with an established migrant protection framework. In addition, the cohort design enables analysis of migration as a prospective measurement and reduces the possibility of recall bias and reverse causation. Furthermore, the fact that data were collected at several points in time allows us to study the effect of age at time of migration. However, the study also has some limitations that should be considered when interpreting its findings. Age at time of migration might be associated with differences in health outcomes, but our small sample size and low statistical power limits our capacity to reject this hypothesis. Due to the small sample size, we were unable to determine if our findings were the same for both girls and boys. Previous research has shown that girls mental health is more likely to be impacted than boys (Jiang et al., 2022; Graham and Jordan, 2011). Sex differences could be culturally specific and could be dependent on which parent migrated. Additionally, by using cash remittances to measure the exposure this study excludes children who experienced paternal emigration but their mother did not receive any financial support from their spouse, which could have impacted the findings as these children are not likely to be improving their SEP and the subsequent benefits that come with that. Moreover, since the CLHNS cohort follows up mothers and their children, we have made the assumption that the spouse of the mother is the father of the child, but this might not always be the case. To try to ensure we were capturing paternal emigration, we limited the sample to children living with their mothers. Children may be living away from their mothers for a number of reasons, including due to both parents migrating, and some exclusions may have underestimated the number children with emigrant fathers. The numbers in this cohort were too small to investigate this further. We may also have missed cases of migration if they occurred between survey intervals. Finally, although we adjusted our analysis for SEP, unmeasured confounding cannot be ruled out and might have altered the observed effects of paternal emigration.

## Conclusion

This study provides preliminary evidence of limited long-term educational and mental health impacts of paternal emigration on children ‘left-behind’. We did not find an association between experiencing paternal emigration and poor mental health outcomes, which may be because we are looking at the long term impacts of the migration experience rather than the short term. It may be that the positive aspects in terms of economic gain and opportunities might mitigate against long-term mental ill health. It is important to determine whether these findings are replicated in other longitudinal data where the support for migrant families is not as well established. Future research could explore whether these associations still hold at older ages. These findings can be useful for policy makers and children's rights activists to develop and advocate for contextual interventions that better support children who experience parental emigration to ensure the positive effects are seen.

## Funding

DK was supported by the Wellcome Trust through an Institutional Strategic Support Fund Award to the University of Bristol (204813/Z/16/Z) and the Elizabeth Blackwell Institute for Health Research University of Bristol. LB was funded by grant MR/W006308/1 for the GW4 BIOMED MRC DTP, awarded to the Universities of Bath, Bristol, Cardiff, and Exeter from the Medical Research Council (MRC)/UKRI. MTR was funded by the National Institute for Health Research (NIHR) Applied Research Collaboration West (ARC West) at University Hospitals Bristol and Weston NHS Foundation Trust (core NIHR infrastructure funded: NIHR200181).

## CRediT authorship contribution statement

**Natalia Norori:** Writing – review & editing, Writing – original draft, Methodology, Formal analysis, Conceptualization. **Lucy Barrass:** Writing – review & editing, Writing – original draft, Formal analysis. **Maria Theresa Redaniel:** Writing – review & editing, Supervision, Methodology. **Nanette R. Lee:** Writing – review & editing, Supervision. **Laura D. Howe:** Writing – review & editing, Supervision, Methodology, Conceptualization. **Duleeka Knipe:** Writing – review & editing, Supervision, Methodology, Data curation, Conceptualization.

## Declaration of competing interest

The authors declare that they have no known competing financial interests or personal relationships that could have appeared to influence the work reported in this paper.

## Appendix Supplementary materials

Image, application 1
