# Supplementary material for: Assessing the impact of paternal emigration on children ‘left-behind’—A cohort analysis
Source: J Migr Health. 2025 Jan 25;11:100308. doi: 10.1016/j.jmh.2025.100308 (PMC11810828; doi:10.1016/j.jmh.2025.100308)

**Supplementary material 1**

**Table S1. Loadings for assets and housing characteristics to the principal component analysis of wealth index**

| **Variable** |  |
| --- | --- |
| **Variance explained by PC1 (%)** | 0.27 |
| Owns air conditioner | 0.19 |
| Owns other appliances | 0.10 |
| Material of house | 0.35 |
| Household size | 0.31 |
| Owns car | 0.23 |
| Owns house | 0.05 |
| Owns TV | 0.46 |
| Owns tape recorder | 0.41 |
| Owns refrigerator | 0.43 |
| Type of household | 0.33 |

**Table S2. Comparison of baseline characteristics between participants included and excluded in the study**

|  | **Included in the Analysis (%)** | **Excluded from the Analysis (%)** | **p value** |
| --- | --- | --- | --- |
| **N=** | 1651 | 1676 |  |
| **Male** | 863 (52.27) | 794 (54.12) | 0.301 |
| **Household type** |  |  | 0.001 |
| One nuclear family | 1073 (64.99) | 999 (59.61) |  |
| **Wealth Index (mean, SD)** | 2.93 (1.41) | 3.03 (1.44) | 0.039 |
| **Paternal education (mean, SD)** | 7.59 (3.85) | 8.12 (3.98) | <0.001 |
| **Experienced paternal emigration at baseline** | 9 (0.55) | 48 (2.86) | <0.001 |

**Figure S1. Flowchart of study population selection, Cebu Longitudinal Health and Nutrition Survey (CLHNS)**


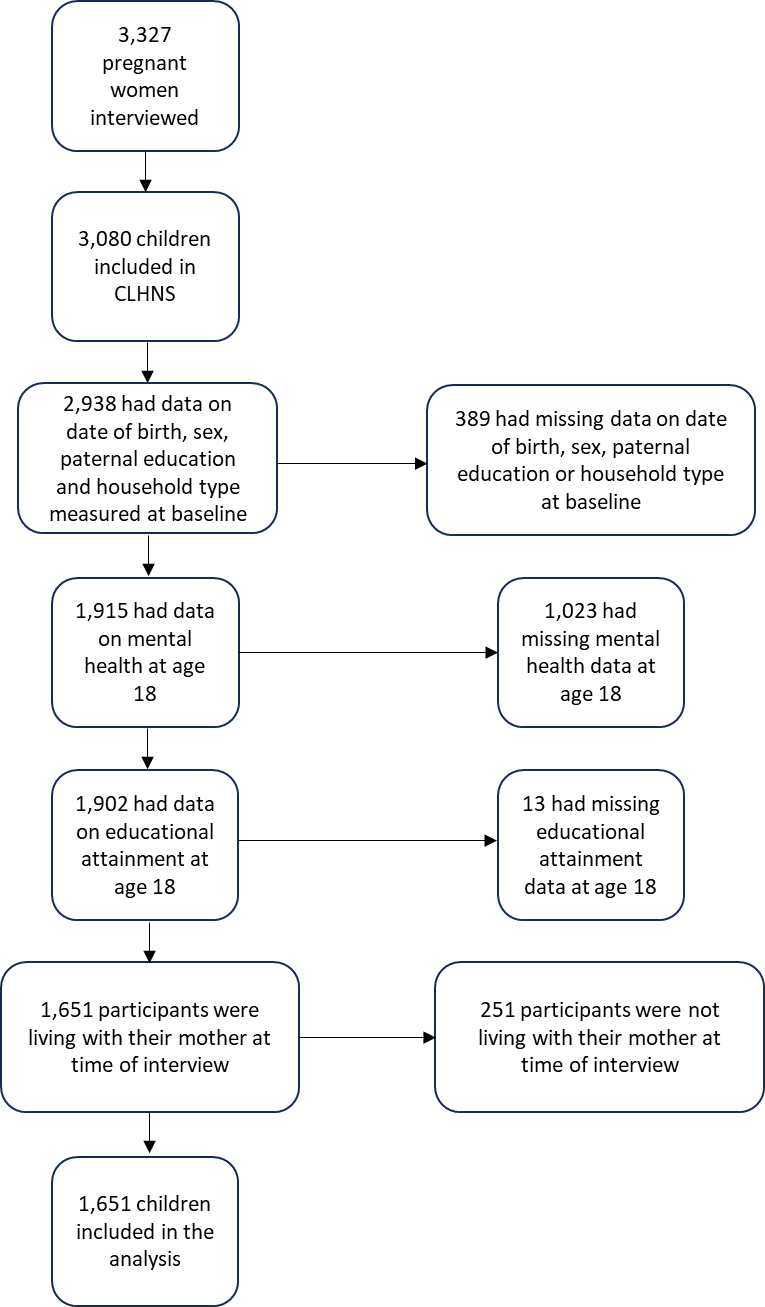

Supplement: Supplementary file 1 [file mmc1.docx]
